# Simulation of macrosegregation in a 2.45-ton steel ingot using a three-phase mixed columnar-equiaxed model^☆^

**DOI:** 10.1016/j.ijheatmasstransfer.2013.08.079

**Published:** 2014-05

**Authors:** Jun Li, Menghuai Wu, Andreas Ludwig, Abdellah Kharicha

**Affiliations:** a Christian Doppler Lab for Adv. Process Simulation of Solidification and Melting, 8700 Leoben, Austria; b Chair of Simulation and Modeling of Metall. Processes, University of Leoben, 8700 Leoben, Austria

**Keywords:** Macrosegregation, Ingot, Multiphase modelling, Crystal sedimentation, Solidification

## Abstract

A three-phase mixed columnar-equiaxed solidification model is used to calculate the macrosegregation in a 2.45 ton steel ingot. The main features of mixed columnar-equiaxed solidification in such an ingot can be quantitatively modelled: growth of columnar dendrite trunks; nucleation, growth and sedimentation of equiaxed crystals; thermosolutal convection of the melt; solute transport by both convection and crystal sedimentation; and the columnar-to-equiaxed transition (CET). The predicted as-cast macrostructure and the segregation pattern are in qualitative agreement with the reported experimental results. Parameter study on the numerical grid size and the nucleation of the equiaxed crystals are performed, and some segregation mechanisms are numerically analyzed. Discontinued positive–negative segregation just below the hot top is predicted because of the formation of a local mini-ingot and the subsequent sedimentation of equiaxed grains within the mini-ingot. Quasi A-segregates in the middle radius region between the casting outer surface and the centreline are also found. The quasi A-segregates originate from the flow instability, but both the appearance of equiaxed crystals and their interaction with the growing columnar dendrite tips significantly strengthen the segregates. The appearance of equiaxed phase is not a necessary condition for the formation of quasi A-segregates. The quantitative discrepancy between the predicted and experimental results is also discussed.

## Nomenclature

*c*_0_: initial concentration, wt.%
$c_{\ell }$, *c*_e_, *c*_c_: species concentration, wt.%
*c*_ref_: reference concentration, wt.%
$c_{\ell }^{*}\text{,}c_{\text{s}}^{*}$: equilibrium concentration at interface, wt.%
$C_{\ell }$_e_: species exchange between liquid and equiaxed, kg Nm^−3^ s^−1^
$C_{\ell }$_c_: species exchange between liquid and columnar, kg m^−3^ s^−1^
*c*_mix_: mix concentration, wt.%
$c_{p}(\ell )$*, c*_p(_*_s_*_)_: specific heat, J kg^−1^ K^−1^
$D_{\ell }$, *D*_e_, *D*_c_: diffusion coefficient, m^2^ s^−1^
*d*_e_: equiaxed grain diameter, m
*d*_c_: columnar trunk diameter, m
$f_{\ell }$, *f*_e_, *f*_c_: volume fraction of different phases, l
$f_{\text{c}}^{\text{free}}$: critical *f_c_* for free moving equiaxed phase, l
$f_{\text{e}}^{\text{c}}$: equiaxed grain packing limit, l
$\overset{\to }{g}$: gravity, m s^−2^
${\overset{\to }{g}}_{\text{e}}^{\prime }\text{,}{\overset{\to }{g}}_{\ell }^{\prime }$: reduced gravity, m s^−2^
*H*: heat transfer coefficient, W m^−2^ K^−1^
*H^∗^*: volume heat transfer coeff. between phases, W m^−3^ K^−1^
$h_{\ell }$, *h*_e_, *h*_c_: enthalpy, J kg^−1^
$h_{\ell }^{\text{ref}}\text{,}h_{\text{e}}^{\text{ref}}\text{,}h_{\text{c}}^{\text{ref}}$: reference enthalpy, J kg^−1^
*K*: permeability of liquid in porous medium, m^2^
$K_{\ell e}(=-K_{e\ell })$: the drag force coefficient, kg m^−3^ s^−1^
*k*: solute partitioning coefficient at the *l*/s interface, l
$k_{\ell }$, *k*_e_, *k*_c_: thermal conductivity, W m^−1^ K^−1^
l: actual columnar length in tip cell, m
$M_{\ell e}(=-M_{e\ell })$: liquid-equiaxed net mass transfer rate, kg m^−3^ s^−1^
$M_{\ell c}(=-M_{c\ell })$: liquid-columnar net mass transfer rate, kg m^−3^ s^−1^
*m*: slope of liquidus in phase diagram, K
*N*_e_: equiaxed grain production rate by nucleation, m^−3^ s^−1^
*n*: equiaxed grain number density, m^−3^
*n*_c_: columnar trunk number density, m^−3^
*n*_max_: maximum equiaxed grain number density, or maximum available nucleation sites in simultaneous nucleation law, m^−3^
*p*: pressure, N m^−2^
$Q_{\ell e}(=-Q_{e\ell }$: total energy exchange between liquid and equiaxed phases, J m^−3^ s^−1^
$Q_{\ell e}^{d}(=-Q_{e\ell }^{d})$: energy transfer between liquid and equiaxed phases, J m^−3^ s^−1^
$Q_{\ell e}^{p}(=-Q_{\mathrm{el}}^{p})$: energy exchange due to phase change between liquid and equiaxed phases, J m^−3^ s^−1^
$Q_{\ell c}(=-Q_{c\ell })$: total energy exchange between liquid and columnar phases, J m^−3^ s^−1^
$Q_{\ell c}^{d}(=-Q_{c\ell }^{d})$: energy transfer between liquid and columnar phases, J m^−3^ s^−1^
$Q_{\ell c}^{p}(=-Q_{c\ell }^{p})$: energy exchange due to phase change between liquid and columnar phases, J m^−3^ s^−1^
*Q*_ec_(=−*Q*_ce_): total energy exchange between equiaxed and columnar phases, J m^−3^ s^−1^
$Q_{\mathrm{ec}}^{d}(=-Q_{\mathrm{ce}}^{d})$: energy transfer between equiaxed and columnar phases, J m^−3^ s^−1^
$Q_{\mathrm{ec}}^{p}(=-Q_{\mathrm{ce}}^{p})$: energy exchange due to phase change between equiaxed and columnar phases, J m^−3^ s^−1^
*R*: radius component of the coordinate, m
*R*(*R*_e_, *R*_c_): grain radius (equiaxed, columnar), m
*R*_f,e_: maximum radius of equiaxed grain, m
*R*_f,c_: maximum radius of columnar trunk, m
*S*_A_: surface area of columnar per volume, m^−1^
*T*_0_: initial temperature, K
*T*, $T_{\ell }$, *T*_e_, *T*_c_: temperature, K
*T_f_*: melting point of pure metal (Fe), K
*T*_ref_: reference temperature for enthalpy definition, K
Δ*T*: constitutional undercooling, K
Δ*T*_N_: undercooling for maximum grain production rate, K
Δ*T*_tip_: undercooling at the columnar dendrite tip, K
Δ*T_σ_*: Gaussian distribution width of nucleation law, K
*t*: time, s
Δ*t*: time step, s
${\overset{\to }{U}}_{\ell e}(=-{\overset{\to }{U}}_{e\ell })$: total liquid-equiaxed momentum exchange rate, kg m^−2^ s^−2^
${\overset{\to }{U}}_{\ell e}^{d}(=-{\overset{\to }{U}}_{e\ell }^{d})$: liquid-equiaxed momentum change due to drag force, kg m^−2^ s^−2^
${\overset{\to }{U}}_{\ell e}^{p}(=-{\overset{\to }{U}}_{e\ell }^{p})$: liquid-equiaxed momentum exchange due to phase change, kg m^−2^ s^−2^
${\overset{\to }{U}}_{\ell c}(=-{\overset{\to }{U}}_{c\ell })$: total liquid-columnar momentum exchange rate, kg m^−2^ s^−2^
${\overset{\to }{U}}_{\ell c}^{d}(=-{\overset{\to }{U}}_{c\ell }^{d})$: liquid-columnar momentum change due to drag force, kg m^−2^ s^−2^
${\overset{\to }{U}}_{\ell c}^{p}(=-{\overset{\to }{U}}_{c\ell }^{p})$: liquid-columnar momentum exchange due to phase change, kg m^−2^ s^−2^
${\overset{\to }{u}}_{\ell }\text{,}{\overset{\to }{u}}_{e}\text{,}{\overset{\to }{u}}_{c}$: velocity vector, m s^−1^
*v*_R_: grain growth speed in radius direction, m s^−1^
*v*_tip_: grain growth speed in tip direction, m s^−1^
*σ*_e_, *σ*_c_: back diffusion length, m
*β*_T_: thermal expansion coefficient, K^−1^
*β*_c_: solutal expansion coefficient, l
$\lambda_{1}$: columnar grain space, m
$\rho_{l}$, *ρ*_e_, *ρ*_c_: density, kg m^−3^
$\rho_{\ell }^{b}$: density for buoyancy force, kg m^−3^
$\mu_{\ell }$, *μ*_e_: viscosity, kg m^−1^ s^−1^
${\overset{¯}{\overset{¯}{\tau }}}_{\ell }\text{,}{\overset{¯}{\overset{¯}{\tau }}}_{e}$: stress-strain tensors, kg m^−1^ s^−1^

**Subscripts**
ℓ: mark liquid
e: equiaxed
c: columnar phases

## 1 Introduction

Macrosegregation, or compositional heterogeneity, is a very common and serious defect in large steel ingots. This compositional heterogeneity occurs due to the relative motion between the liquid and solid phases during solidification. This relative motion can arise from thermosolutal convection, shrinkage-induced feeding flow, flotation and sedimentation of free moving crystals, mechanical or electromagnetic stirring, flow induced by pore or gas bubble formation, deformation of the solid skeleton, and capillary force-induced flow [1]. A lot of experiments were done to study the macrosegregation in steel ingots [2–4]. The typical segregation pattern in a steel ingot consists of a positive segregation in the upper region, a conic negative segregation in the lower region, V-segregates along the centreline, and A-segregates in the middle radius region between the casting outer surface and the centreline [5,6].

Since the first modelling attempt by Flemings et al. [7,8], several macrosegregation models have been developed [9–12]. Gu and Beckermann [12] were the first to apply a coupled multicomponent solidification model with melt convection to a large industry-scale ingot (1.016 × 2.08 × 2.819 m^3^); their simulation qualitatively agreed with the positive segregation observed in the upper region of the ingot. However, because the sedimentation of free equiaxed crystals was neglected, they could not properly predict the negative segregation at the lower region of the ingot. Ludwig et al. [13] presented a new method of coupling a ternary phase diagram with a multiphase process simulation. Their model was applied to a continuous bronze casting, and a reasonable result was obtained. Combeau et al. [14,15] presented a two-phase (solid and liquid) model to study the influences of both motion and morphology of equiaxed grains on a 3.3-ton steel ingot. Some progress was made towards predicting the behaviour at the negative segregation zone in the lower region of the ingot, and this model is in good agreement with the experimental results. However, these researchers did not distinguish the columnar phase from the equiaxed phase; both of which are present and interact with each other during solidification.

Researchers have been investigating the mechanism of formation of A-segregation (channel segregation) for decades, and various mechanisms have been suggested [4,16,17]. The most accepted mechanism supports that the main factor in producing A-segregation is the flow of the segregated melt within the mushy zone, which is primarily controlled by both the alloy composition and the thermal conditions prevailing during the solidification process.

Wu et al. [18,19] developed a mixed columnar-equiaxed solidification model, which directly accounts for the nucleation and growth of equiaxed globular grains, the growth of columnar dendrite trunks, and the effect of both equiaxed grain sedimentation and melt convection. The previous studies successfully predicted both the conic negative segregation in the lower region of the ingot and the columnar-to-equiaxed-transition (CET). However, due to the limited computational capability, the previous studies were only based on the laboratorial scale of steel ingots. In this study, the previous model is modified to study macrosegregation in a 2.45-ton, large-end-up, industry steel ingot that was reported in the literature [3].

## 2 Model description

Details of the three-phase mixed columnar-equiaxed solidification model can be found in the literature [18–22]. Here, only a brief outline of the model is given.

• Three phases are defined: the primary liquid phase (l), the equiaxed phase (e), and the columnar phase (c). The corresponding phase fractions are $f_{l}$, *f*_e_ and *f*_c_ with $f_{l}$ + *f*_e_ + *f*_c_ = 1. Both the liquid and equiaxed phases are moving phases, for which the corresponding Navier–Stokes equations are solved. The columnar phase is assumed to adhere to the wall, thus a momentum equation for the columnar phase is not considered.
• Columnar dendrites are approximated by cylinders extending from the mould wall towards the casting centre. The advance of the columnar tip front is tracked during the solidification.
• Equiaxed grains are approximated as spheres. However, to calculate the drag force, the morphology of the equiaxed grains is modelled as octahedra.
• A three-parameter heterogeneous nucleation law is used for the nucleation of equiaxed grains. Neither fragmentation nor grain attachment is currently considered.
• The growth of both the columnar trunk and the equiaxed grain are governed by diffusion; the concentration difference $\left(c_{l}^{*}-c_{l}\right)$ is the driving force for the growth of columnar trunks and equiaxed grains. Back diffusion in the solid is also considered for both equiaxed and columnar phases.
• Volume-averaged concentrations $\left(c_{l}\text{,}c_{s}\right)$ are calculated, and macrosegregation is characterised by the concentration of the mixture, *c*_mix_. Thermodynamic equilibrium is assumed at the liquid–solid interface, which dictates the concentrations at the liquid–solid interface $\left(c_{l}^{*}\text{,}c_{s}^{*}\right)$.
• Enthalpy equations for all three phases are solved. Due to the fact of local thermal equilibrium, it is only necessary to have one temperature representing all phases in each volume element. Therefore, a large volumetric inter-phase heat transfer coefficient *H*^∗^ is applied to level out the temperature difference among phases. *H*^∗^ should be set as large as possible, but a too large value of *H*^∗^ leads to divergence of the calculation. The value recommended in Table 1 (10^9^ W m^−2^ K^−1^) can fulfil a condition of the calculated temperature difference between phases less than 10^−3^ K, and in the meantime the numerical calculation can fulfil the corresponding convergence criterion.
• A linearized, binary Fe–C phase diagram is used with a constant solute partition coefficient *k* and a constant liquidus slope *m*.
• Resistance to interdendritic flow in the mushy zone is calculated using a permeability law based on the Blake–Kozeny approach [23].
• The growth of the columnar primary tips stops when the volume fraction of the equiaxed phase reaches 0.49 (hard blocking criterion) in front of the primary columnar dendrite tips [24].
• The packing limit for the equiaxed phase is set as *f*_e_ + *f*_c_ ⩾ 0.637 [18,19,25,26], and the equiaxed crystals are trapped by the columnar dendrite trunk when the local volume fraction of the columnar phase reaches a critical value of 0.2 [19,26].
• Solidification shrinkage is not accounted for, and thermosolutal convection is modelled using the Boussinesq approach.

Conservation equations, sources and exchange terms, and auxiliary equations are summarised in Table 1. The symbols used in the table are described in the nomenclature.

The model described here is developed within the framework of the CFD software package, ANSYS-Fluent version 14.5, which provides a platform for solving the conservation equations and provides flexibility in defining exchange and source terms within the conservation equations, including modification of the transport quantities. For each time step, up to 60 iterations may be necessary to decrease the normalised residuals of continuity, momentum conservation, volume fraction, species transport and user-defined scalar conservation equations to a value below the convergence criterion of 10^−4^ and the enthalpy conservation equations below that of 10^−7^. Due to the complexity of the multiphase coupling, the time step should be kept small (10^−2^–10^−3^) to ensure the above convergence criteria are fulfilled. The optimal time step must be determined by trial simulations. All calculations are run in parallel on 8 CPUs (Intel Nehalem Cluster 2.93 GHz).

## 3 Configuration of the ingot

**Fig. 1:**
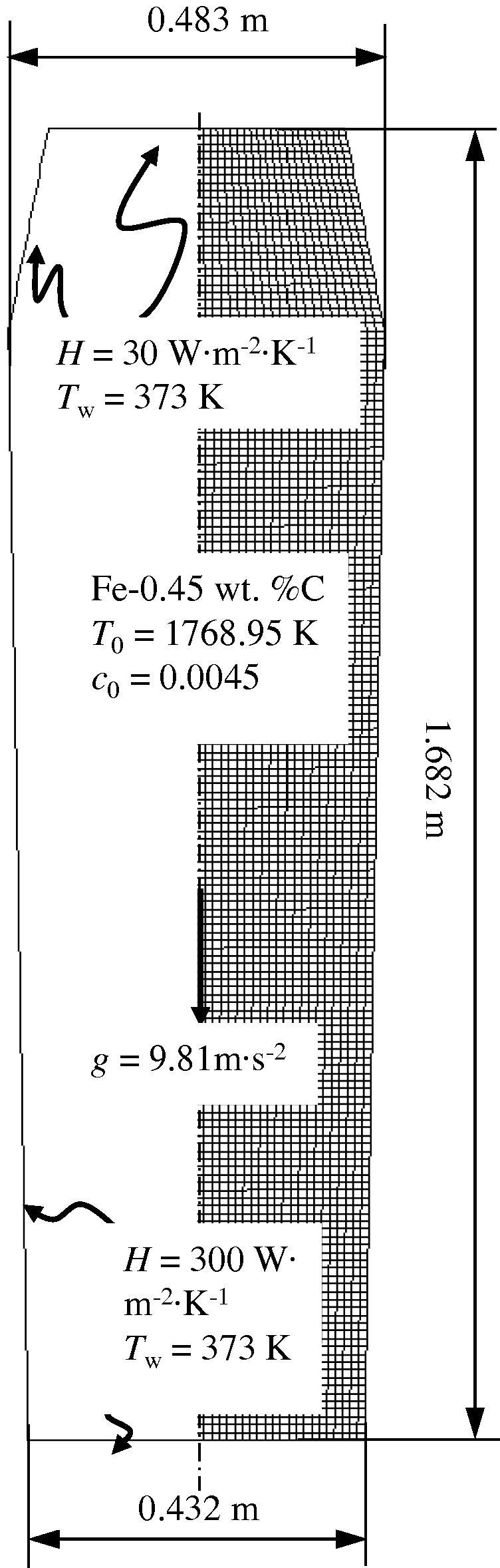
Configuration of the 2.45-ton industry steel ingot.

A large number of industry steel ingots have been poured and analysed for macrosegregation during the last century [2,3]. They were sectioned longitudinally along the axes, and subject to two different analyses: sulphur print and chemical analysis of drilling samples. The sulphur print, presenting the degree of heterogeneity of the sulphur element, was taken as indicative of general heterogeneity of other elements like carbon and phosphorus. Chemical analysis of drilling samples (average diameter of 10 mm drilled from the ingot section) gave a quantitative macrosegregation map for each alloy element. Here a representative 2.45-ton large-end-up ingot (Fe–0.45 wt.%C) is studied. The ingot had a square, cross-sectional area and was cast in a chilled mould; a 2D-axis symmetrical simulation was performed to approximate the solidification behaviour in the square cross-section of the ingot. The configuration of this ingot, along with the necessary boundary and initial conditions, is described in Fig. 1. Table 2 lists the thermodynamic and physical properties of the ingot. Because the experiment was performed many decades ago and because of the lack of a precise process description, assumptions must be made for certain process parameters and boundary conditions. Here, the heat transfer coefficient is based on the final solidification time, which refers to the classical theory and experimental data [2].

## 4 Simulation result

### 4.1 Solidification sequence

**Fig. 2:**
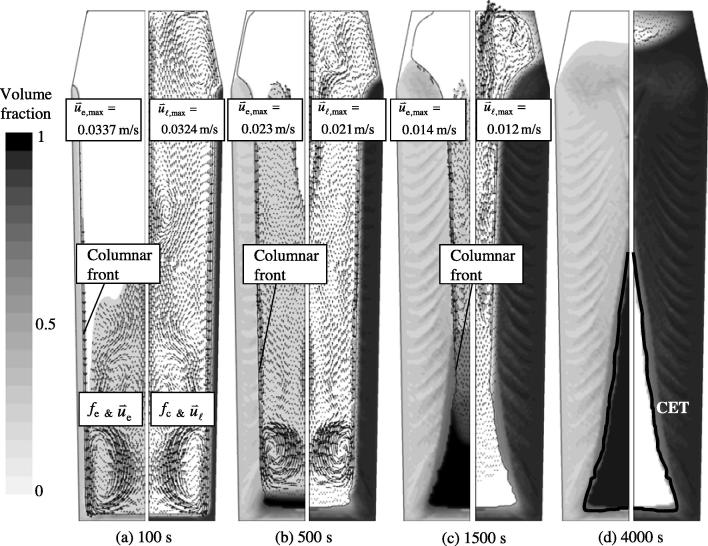
The predicted solidification sequence of the 2.45-ton ingot. The volume fraction of each phase (*f*_e_ or *f*_c_) is shown in grey map with 20 grey levels from 0 (white) to 1 (black). The left half of each graphic shows the evolution of the equiaxed volume fraction (*f*_e_) and the equiaxed sedimentation velocity $\left({\overset{\to }{u}}_{\text{e}}\right)$, indicated by the black arrows. The right half of each graphic shows the evolution of the columnar volume fraction (*f*_c_) and the melt velocity $\left({\overset{\to }{u}}_{l}\right)$, indicated by the black arrows. We did not show velocity vectors for each numerical grid point but rather selected representative vectors. The position of the columnar dendrite tip was marked with a black solid line. The nucleation parameters for this calculation are *n*_max_ = 2 × 10^9^ m^−3^ and Δ*T_σ_* = 2 K, Δ*T*_N_ = 5 K; the numerical grid size is 5 mm.

The solidification sequence is shown in Fig. 2 and it includes the sedimentation of equiaxed crystals, the sedimentation-induced and thermosolutal buoyancy-induced melt convections. The simulated solidification sequence agrees with the theoretical explanation of steel ingot solidification, as summarised by Campbell [30]. Columnar dendrites grow from the mould wall, and the columnar tip front moves inwards; equiaxed grains nucleate near the mould walls. Columnar dendrites are stationary, whereas equiaxed grains sink and settle at the base of the ingot. The accumulation of such grains at the base of the ingot results in a characteristic conic shape. Both the sedimentation of grains and the melt convection influence the global solidification sequence and, thus, the final phase distribution. More equiaxed grains will be found in the lower region of the ingot, whereas columnar structures will be predominant in the upper region of the ingot.

The flow in the ingot is driven by three forces: the solutal buoyancy force, which leads to upward flow; the thermal buoyancy force, which leads to downward melt flow; and the motion of the sinking equiaxed crystals, which drags the surrounding melt downwards. Initially, the two downward forces are dominant along the side walls of the ingot. The downward flow and the sinking equiaxed crystals change direction at the bottom of the ingot, move inwards, and then rise in the middle region of the ingot. Meanwhile, the cooled melt at the casting top tends to sink directly from the middle part of the ingot. The upward and downward flows interact with each other, and the resulting global flow pattern is highly unstable and disordered. Large eddies develop dynamically and then are suppressed in the bulk region. Due to the interactions between the sinking equiaxed crystals and the growing columnar tip fronts in the melt, some small convection cells can develop along the columnar tip front. These small convection cells travel with the global flow current, integrate into the global flow current and then may finally disappear in the bulk.

In the middle region of the ingot, columnar tip fronts from both sides meet at the ingot centre during the late stage of solidification. In the lower part of the ingot, however, the large amount of equiaxed grains settle there, and stop the propagation of the columnar tip front. The final position of the columnar tip front, which is blocked by the equiaxed crystals, indicates the columnar-to-equiaxed transition (CET), as shown in Fig. 2(d). The CET line separates the areas where only equiaxed grains appear from the areas where both columnar dendrites and equiaxed grains coexist.

Additionally, due to the complexity of the flow pattern and the interaction among the melt, equiaxed and columnar phases, a streak-like distribution pattern between the columnar and equiaxed phases is gradually developed within a specific distance from the side walls. This streak-like phase distribution ultimately affects the final macrosegregation.

**Fig. 3:**
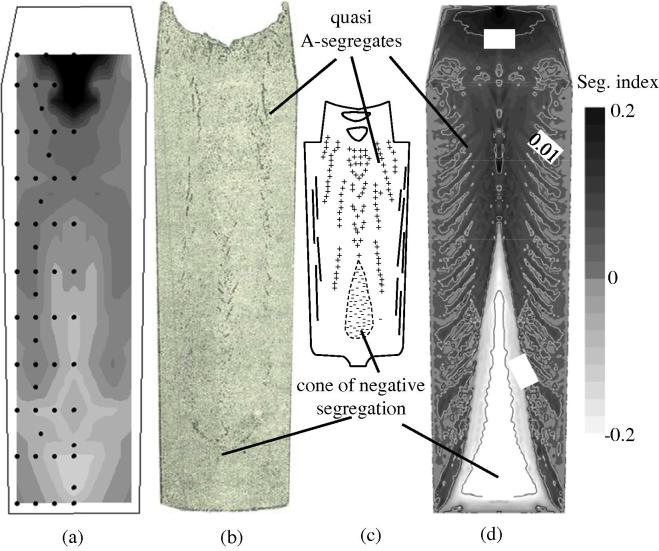
Macrosegregation pattern. (a) Reconstructed segregation map in grey scale out of chemical analysis of 54 drilling samples, (b) sulphur print of the as-cast ingot [3], (c) schematic of the typical macrosegregation pattern in steel ingots [16], and (d) predicted macrosegregation pattern (black for the positive segregation and white for the negative segregation) overlapped with isolines. The segregation is quantified by a segregation index: (*c*_mix_ − *c*_0_)/*c*_0_.

Fig. 3 shows the comparison of the predicted segregation result with experimental one [3]. The measured carbon segregation map in grey scale is reconstructed from the chemical analysis of 54 drilling samples. The sulphur print gives a qualitative indication of general segregation pattern. In this paper, the macrosegregation was quantified by a segregation index: (*c*_mix_ − *c*_0_)/*c*_0_. A positive segregation in the top and a conic negative segregation in the bottom were observed. Obviously, the lower conic negative segregation is caused by the equiaxed sedimentation. The settling grains are poor in solute elements, thus their accumulation results in negative segregation. The positive segregation at the top region of the ingot is caused by the flow of the enriched melt in the bulk region. A streak-like segregation pattern in the middle radius region between the outer surface and the ingot centreline is predicted. One may notice that this streak-like segregation has a similar contour to the classical A-segregation (Fig. 3(c)), but the current paper cannot justify if the classical A-segregation is the same as streak-like segregation or originates from this type of streak-like segregation. According to the most widely accepted empirical explanation [16], A-segregation belongs to a type of channel segregation in large steel ingots, which originates and develops in the stationary dendritic mushy zone. Therefore, we referred to the streak-like segregations as quasi A-segregates (or quasi A-segregation). Further discussion about the formation mechanism of the quasi A-segregates can be found in Section 6.2.

### 4.2 Experimental verification

**Fig. 4:**
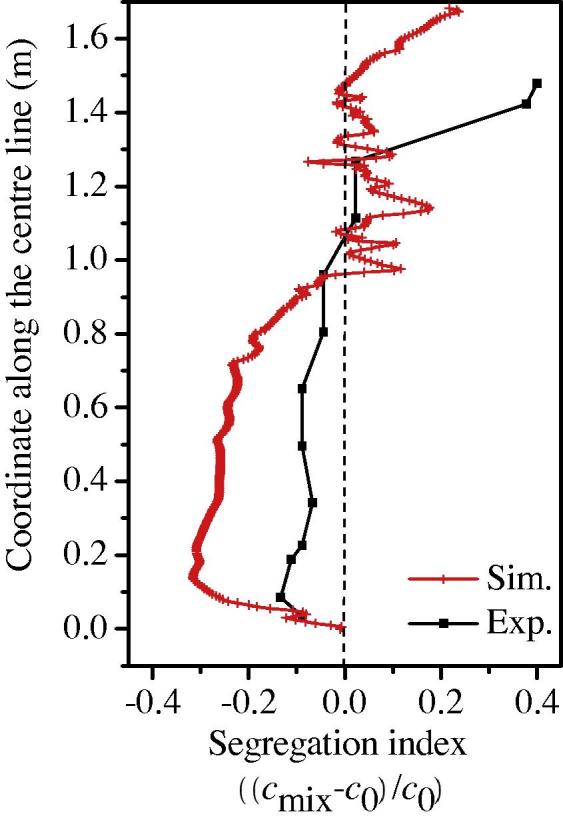
The comparison result of the centreline of the segregation index.

The predicted macrosegregation pattern was compared with the sulphur print and measured segregation pattern, as shown in Fig. 3. Both the conic negative segregation in the lower region of the ingot and the quasi A-segregation bands were predicted. This pattern coincides qualitatively with the experiment result and fit to the typical segregation pattern of steel ingots. The segregation along the ingot centreline is compared with the experiment report [3], as shown in Fig. 4. Both the experimental and simulation results show the negative segregation in the lower part and positive segregation in the upper part. However, the negative segregation in the lower part is predicted more severely than the experimental result. The overestimation of the negative segregation in the lower part by the model results from two aspects. One is the assumption of globular equiaxed morphology, which can cause significant overestimation of the sedimentation-induced negative segregation. The other aspect is the lack of real process conditions and material property, especially in terms of the parameters for the nucleation of equiaxed crystals.

In the upper part of the ingot, the experiment result shows a large positive segregation in the hot top. The modelling result shows also a large positive segregation in the hot top, but a discontinuous distribution between negative and positive segregation can be identified just below the hot top. The predicted position of the hot top is higher than the experimentally indicated position. The reasons for the above discrepancy are from both experimental and modelling aspects. From the modelling aspect, the uncertainty about the thermal boundary conditions and the neglect of the formation of the shrinkage cavity in the top are mainly responsible for the discrepancy between the predicted and experimentally indicated hot top positions. From the experiment aspect, the distance between two sampling points is larger than 10 cm, and it might not provide sufficient measurement resolution. In the practise, people often identified the discontinuous negative-positive segregation pattern below the hot top [14]. The reason for this type of segregation distribution is due to the formation of mini-ingot in the late stage of solidification. Further discussion of the mini-ingotism is made in Section 6.1.

One should emphasise that a main motivation of the current study is to verify the capability of the mixed columnar-equiaxed solidification model. This study is not going to adjust the process parameters to cater for the experiment results. Because the experiments were carried out decades ago and many of the process parameters and material properties were not reported, the current simulation results could only qualitatively reproduce the experiment results.

## 5 Parameters study

### 5.1 Grid size

**Fig. 5:**
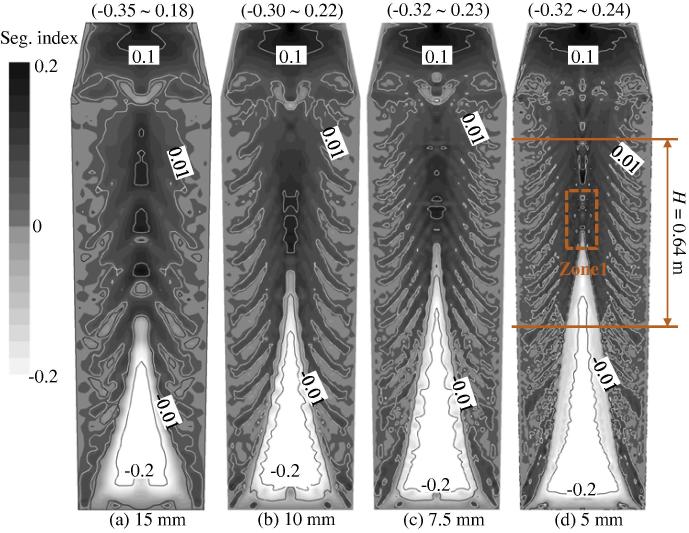
Grid dependency of the macrosegregation map. The segregation index is shown in greyscale from −0.2 (white) to 0.2 (black), overlapped by its isolines. The calculations were made using equiaxed nucleation parameters: *n*_max_ = 2 × 10^9^ m^−3^, Δ*T_σ_* = 2 K, and Δ*T*_N_ = 5 K.

**Fig. 6:**
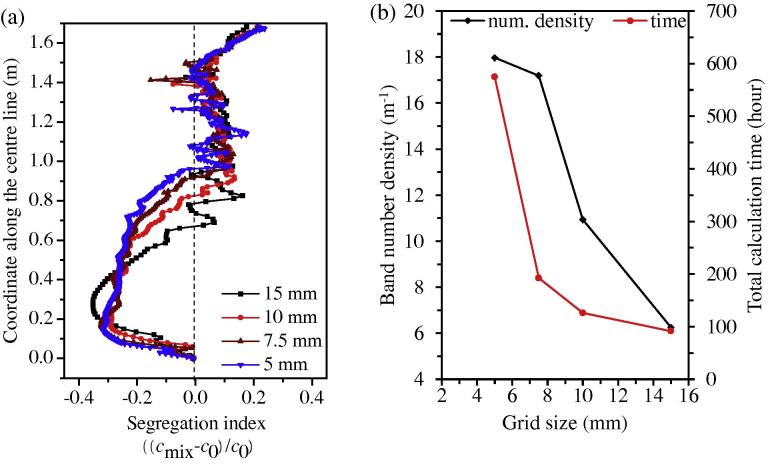
(a) Grid dependency of the segregation profile along the ingot centreline and (b) grid dependency of the band number density and calculation time. The calculations are run in parallel on a Cluster (Intel Nehalem Cluster 2.93 GHz) with 8 CPU cores.

Calculations with different grid sizes were performed, and the segregation results are shown in Figs. 5 and 6. As the grid size increases from 5 mm to 15 mm, a similar global segregation pattern can be predicted: positive macrosegregation at the hot top, a conic negative segregation in the lower region and some quasi-A segregation bands in the middle radius region between the casting outer surface and the centre line. The positive segregation extreme of approximately 0.22 to 0.24, located in the hot top, and the negative segregation extreme of about −0.30 to −0.32, located in the lower equiaxed sedimentation zone, do not significantly change when the grid size is less than 10 mm. The height of the lower, negatively-segregated sedimentation zone increases slightly with the decrease of the grid size, but this change is not significant when the grid size is less than 7.5 mm. We can conclude that for the current 2.45-ton ingot, a grid size of 7.5–10 mm is sufficient to attain a global segregation pattern. Because the calculation cost (Fig. 6(b)) increases exponentially with grid resolution, a relatively coarse grid (e.g., 10 mm) is recommended to obtain a global macrosegregation pattern.

The quasi A-segregation is more sensitive to grid size. A band number density, *N*/*H* (1/m), is defined to investigate the grid dependency of quasi A-segregation. *N* is the number of quasi-A segregation bands in the range of *H*, as indicated in Fig. 5. The band number density increases with an increase in the grid resolution, as shown in Fig. 6(b). The *N*/*H* curve is prone to become constant when the grid size is smaller than 5 mm, thereby suggesting that the grid-independent result of the *N*/*H* ratio might be achieved. However, the fine details of each quasi A-segregate are still not convergent. An absolute grid-independent result with respect to the fine details of quasi A-segregates has not yet been achieved with a grid size of 5 mm. The calculation time increases exponentially with the increase of the grid resolution.

### 5.2 The nucleation parameters

**Fig. 7:**
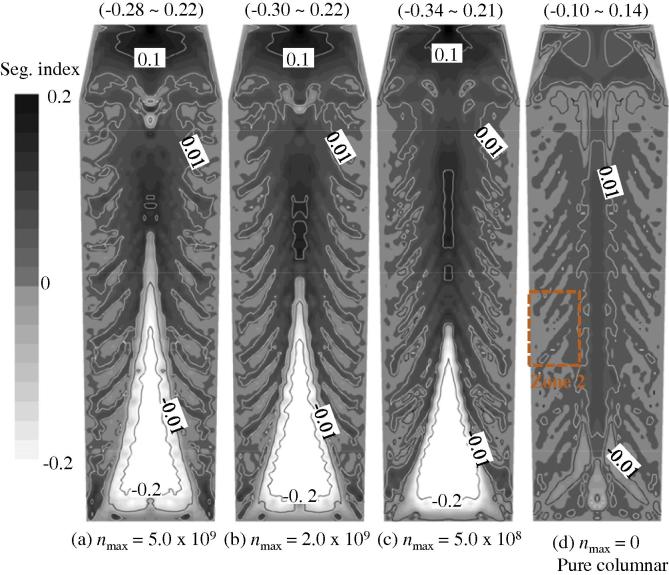
Influence of *n*_max_ on the macrosegregation map. The segregation index is shown in greyscale from −0.2 (white) to 0.2 (black), overlapped by its isolines. The calculations were made using a grid size of 10 mm; the other nucleation parameters, Δ*T_σ_* = 2 K and Δ*T*_N_ = 5 K, were constant.

**Fig. 8:**
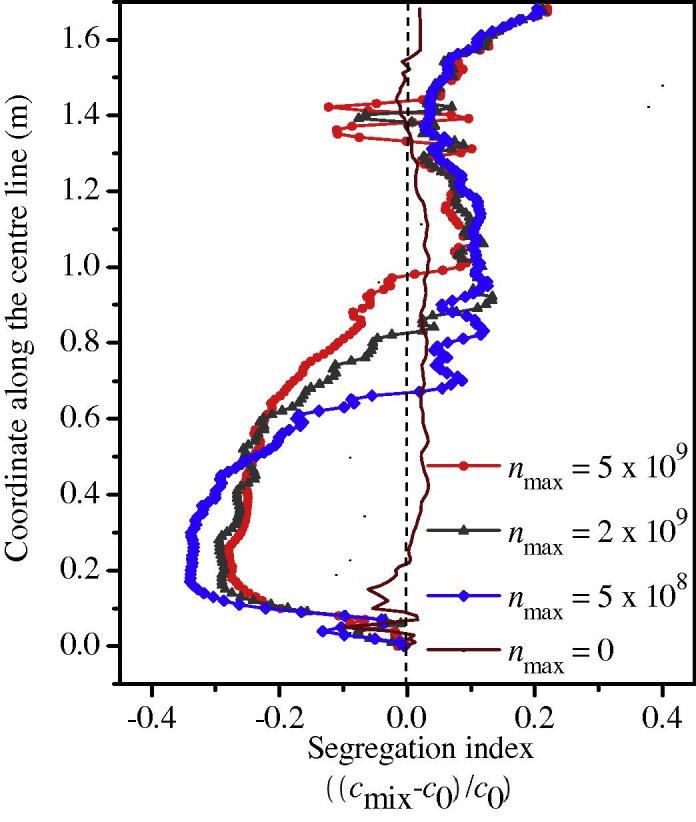
Influence of *n*_max_ on the centreline segregation profile. Calculations were made using a grid size of 10 mm; the other nucleation parameters, Δ*T_σ_* = 2 K and Δ*T*_N_ = 5 K, were constant.

**Fig. 9:**
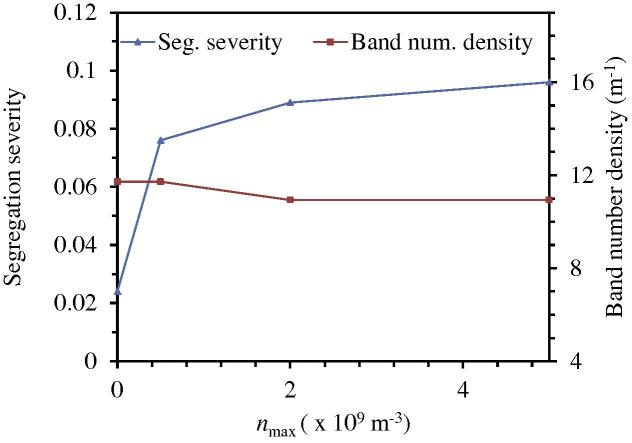
Influence of *n*_max_ on the segregation severity across the quasi A-segregation band $(c_{\text{mix}}^{\text{max}}-c_{\text{mix}}^{\text{min}})/c_{0}$ and band number density. Calculations were made with a grid size of 10 mm; the other nucleation parameters, Δ*T_σ_* = 2 K and Δ*T*_N_ = 5 K, were constant.

A three-parameter law (Eq. (13)) is used to model the nucleation of equiaxed crystals; in this study, only one parameter, *n*_max_, is varied from 0 to 5 × 10^9^ m^−3^, while the other two, Δ*T_σ_* = 2 K and Δ*T*_N_ = 5 K, are kept constant. The final results are shown in Figs. 7–9. The case with *n*_max_ = 0 corresponds to pure columnar solidification.

The most significant influence of *n*_max_ is on the bottom sedimentation-induced negative segregation zone. The height of the conic zone decreases with decreasing *n*_max_. For pure columnar solidification, this zone disappears. With respect to the global segregation severity (the extent of variation between the positive segregation extreme and the negative segregation extreme), there is a large difference between mixed columnar-equiaxed solidification and pure columnar solidification. As shown in Fig. 7 for pure columnar solidification, the maximum positive segregation is 0.14 and the negative segregation extreme is −0.10. By contrast, for mixed columnar-equiaxed solidification, the maximum positive segregation is approximately 0.21–0.22 and the negative segregation extreme is about −0.28 to −0.34. There are two reasons for the differences in segregation behaviour: sedimentation of the equiaxed crystals creates a large amount of negative segregation in the lower segregation zone and this sedimentation induces much stronger melt convection in the bulk region. Thus, sedimentation of crystals is a very important mechanism responsible for global macrosegregation.

Upon comparison of the three cases of mixed columnar-equiaxed solidification (Fig. 7(a)–(c)), we find that the global segregation patterns are quite similar. The maximum positive segregation in the hot top (0.21–0.22) seems to be insensitive to variations in *n*_max_ from 5 × 10^8^ m^−3^ to 5 × 10^9^ m^−3^. The negative segregation extreme in the sedimentation zone becomes slightly more negative with decreasing *n*_max_ values (Fig. 8). The reason is that the average grain size of equiaxed crystals (*d_e_*) is predicted to increase with the decrease of *n*_max_ The drag force of the surrounding melt acting on the settling equiaxed crystals decreases with the grain size, i.e. ${\overset{\to }{U}}_{l\text{e}}^{\text{d}}\;\alpha \;d_{\text{e}}^{-2}$ [21]. Therefore, the settling velocity of the equiaxed crystals increases with the decrease of *n*_max_, hence negative segregation extreme in the sedimentation zone becomes slightly more negative.

In Fig. 7, the quasi A-segregation patterns look similar for all cases. The general quasi A-segregation bands (e.g., the band number density) are almost the same. As shown in Fig. 9, the band number density is independent of *n*_max_. The main difference is the segregation severity across the band space. Here, a new quantity is defined to analyse the segregation severity across the quasi A-segregation band, $\left(c_{\text{mix}}^{\text{max}}-c_{\text{mix}}^{\text{min}}\right)$, where $c_{\text{mix}}^{\text{max}}$ is the maximum *c*_mix_ and $c_{\text{mix}}^{\text{min}}$ is the minimum *c*_mix_ across the band space. These two values are actually read from a representative band in the middle of Zone 2 as marked in Fig. 7(d). As observed in Fig. 9, the segregation severity across the quasi A-segregation band for the cases of mixed columnar-equiaxed solidification is notably larger than for the case of pure columnar solidification. Therefore, the appearance of equiaxed crystals in such a steel ingot strengthens the segregation severity across the quasi A-segregation band.

## 6 Discussion

### 6.1 Bridging and mini-ingotism

The discontinuous negative-positive segregation below the hot top results from the formation of a local mini-ingot. Bridging and mini-ingotism are phenomena that were first reported in continuously cast round billets of steel [31]. The ingot used in this study is quite long, and solidification in such a long ingot behaves similarly to that in a continuously cast round billet. At later stage of solidification, because the middle part of the ingot is already blocked by the columnar trunks, the solidification of the remaining melt in the upper part behaves like the solidification of a mini-ingot. This local mini-ingot is observed as a long, narrow, and large-end-up tube. The equiaxed grains sediment from the upper part to the lower part and then accumulate in the lower region of the mini-ingot.

**Fig. 10:**
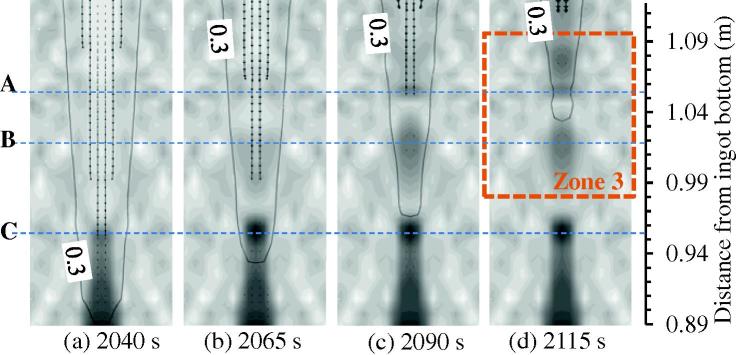
The solidification sequence at the bottom of the mini-ingot, as viewed in Zone 1 (marked in Fig. 5(d)). The equiaxed grain number density is shown in greyscale from a minimum value of 10^8^ m^−3^ to a maximum value of 8 × 10^8^ m^−3^. The isoline of $f_{l}=0.3$ is also shown. The vectors indicate the velocity of the settling equiaxed crystals.

**Fig. 11:**
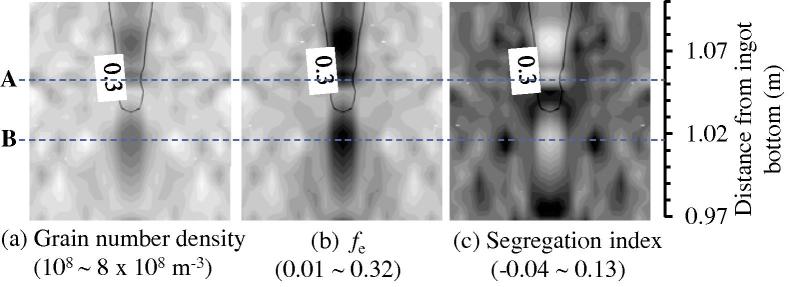
Zoom-in result in Zone 3 (marked in Fig. 10(d)) at 2115 s. All quantities are shown in greyscale with maximum and minimum values given. The isoline of $f_{l}=0.3$ is also overlaid on this figure.

Fig. 10 shows the evolution of the equiaxed grain number density and the velocity field of the crystal sedimentation in the lower region of the mini-ingot. There are several locations, some of which are marked with symbols A through C in Fig. 10, where the equiaxed grain number density increases much faster than it does in the neighbouring regions. Correspondingly, the volume fraction of the equiaxed phase in these locations is larger than in the neighbouring regions, as shown in more detail in Fig. 11. It is anticipated that the equiaxed crystals accumulate there due to sedimentation and accumulate relatively isolated islands of the equiaxed phase.

**Fig. 12:**
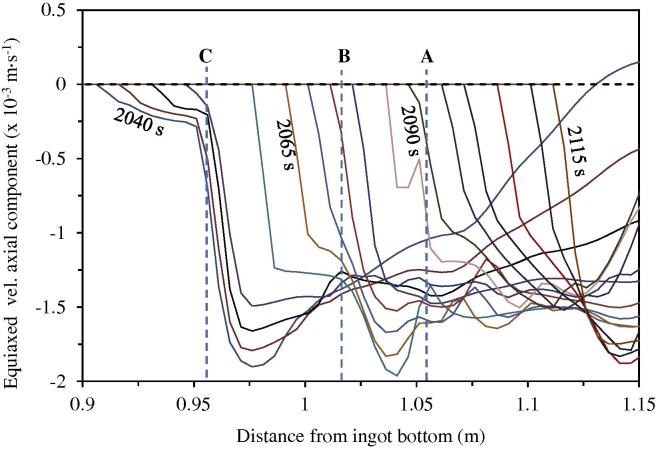
Evolution of the axial component of the equiaxed velocity along the casting centreline. The velocity curves are plotted with equal time interval of 5 s. The corresponding locations (A through C) are also indicated, as marked in Fig. 10.

Fig. 12 plots the evolution of the axial component of the equiaxed velocity along the casting centreline. This finding provides more clear evidence about the crystal accumulation. The negative value of the velocity component means that the crystals sink downwards. The variation in the velocity curves along the centreline at different moments is not monotonic. If the magnitude of the velocity component suddenly decreases at a given location, the local accumulation of the crystals occurs. Correlating the velocity curves in Fig. 12 with the accumulation locations (A through C) in Fig. 10, we find that just above this accumulation site, there is a clear indication of the reduction in velocity.

The accumulation of the equiaxed phase in those islands induces negative segregation, as shown in Fig. 11(c). The mechanism for this negative segregation is the same as the conic negative segregation in the base region of the ingot. The discontinuous distribution between the negative (islands) and positive segregation below the hot top is due to the interaction between the growing columnar tip front and the accumulation of the equiaxed phase. If the columnar tip front blocks the casting centre again inside the mini-ingot, a new mini-ingot would form. This process may repeat several times, depending on the casting geometry and the cooling boundary conditions of the hot top.

It is interesting to observe that the discontinuous distribution between the negative and positive segregation follows a similar pattern of V-segregates, as seen in Figs. 5 and 11. It implies that the formation of mini-ingot and the sedimentation of equiaxed crystals at the bottom of the mini-ingot might contribute to the formation of V-segregates. In addition to its similar appearance to a V-shape, the position where the segregate forms and the thermal conditions under which it forms follow the classical elucidation of V-segregates. However, this hypothesis needs further investigation. As we also learnt [16,31,32] that some other important factors influencing V-segregates, e.g. the sucking flow due to solidification shrinkage and the deformation of the dendrites in mushy zone along the casting centreline, are not taken into account in the current model.

### 6.2 Quasi A-segregation

A recent study by the authors [33] in a Sn-Pb laboratory casting has found that channel segregation can originate and develop during pure columnar solidification, in which no equiaxed crystals exist. Channel segregation develops in two steps: (1) the initiation of the channel, caused by flow instability, and (2) the growth of the channel, caused by the flow-solidification interaction. Remelting is not necessary for formation of the channels. Actually, the quasi A-segregates belong to the same channel segregates as those studied in the previous work [33]. The term ‘quasi A-segregates’ is defined here, distinct from the classical term ‘A-segregates’, because it was previously believed that remelting was the key mechanism for formation of A-segregates. However, neither the current work nor the previous work [33] found clear evidence of remelting. Nevertheless, streak-like channel segregates occur.

A parameter study on the nucleation of equiaxed crystals, in Figs. 7 and 9, seems to show that the quasi A-segregation pattern is independent of the appearance of equiaxed phases. Both the three-phase mixed columnar-equiaxed solidification model and the two-phase columnar solidification model predict a similar pattern of quasi A-segregates. This result leads to a hypothesis that the appearance of an equiaxed phase is not a necessary condition for the formation of quasi A-segregates and that the cause of quasi A-segregates is the same in both solidification cases, i.e., it is initiated by flow instability. However, Figs. 7 and 9 show that the segregation severity across each quasi A-segregation band is strengthened by the equiaxed phase. Two possible reasons are discussed as follows.

**Fig. 13:**
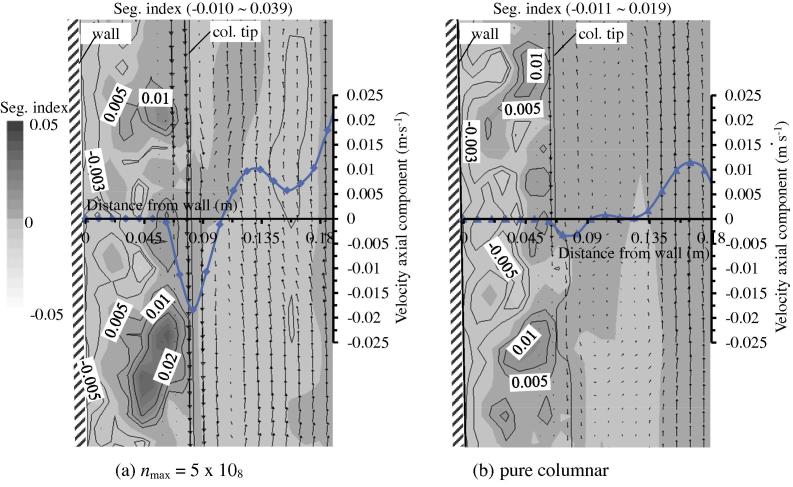
Comparison of the two solidification cases at the initial stage of quasi A-segregate formation: (a) mixed columnar-equiaxed solidification (*n*_max_ = 5 × 10^8^ m^−3^) and (b) pure columnar solidification. The figures show the zoomed-in results in Zone 2 (marked in Fig. 7(d)) at 400 s. The segregation index is shown in greyscale overlapped by its isolines, which denote the initial stage of quasi-A segregation. The melt flow is shown in vectors. Additionally, the axial component of the equiaxed grain sedimentation is plotted along a horizontal line. The position of the columnar tip front is indicated.

Firstly, the appearance of equiaxed phase accelerates the melt flow in the vicinity of the columnar tip front where the quasi A-segregates initiate and develop. Fig. 13 compares the melt flow at the initial stage (400 s) of quasi A-segregate formation between the two solidification cases: mixed columnar-equiaxed solidification and pure columnar solidification. Although the quasi A-segregation patterns in both cases are quite similar, they are not quantitatively identical. The magnitude of velocity during mixed columnar-equiaxed solidification is much (approximately 4 times) larger than that during pure columnar solidification. Sedimentation of equiaxed crystals accelerates the flow in the surrounding melt. Ultimately, the accelerated melt flow aggravates the severity of segregates. From Fig. 7 it is also noticed that the incline angle of the quasi A-segregates is slightly influenced by the accelerated flow due to equiaxed sedimentation. The segregation bands in the cases of mixed columnar-equiaxed solidification ‘bent’ more towards the top of ingot than in the case of pure columnar solidification. This phenomenon was discussed previously by the current authors [33]. The incline angle increases with the intensity of the interdendritic flow.

**Fig. 14:**
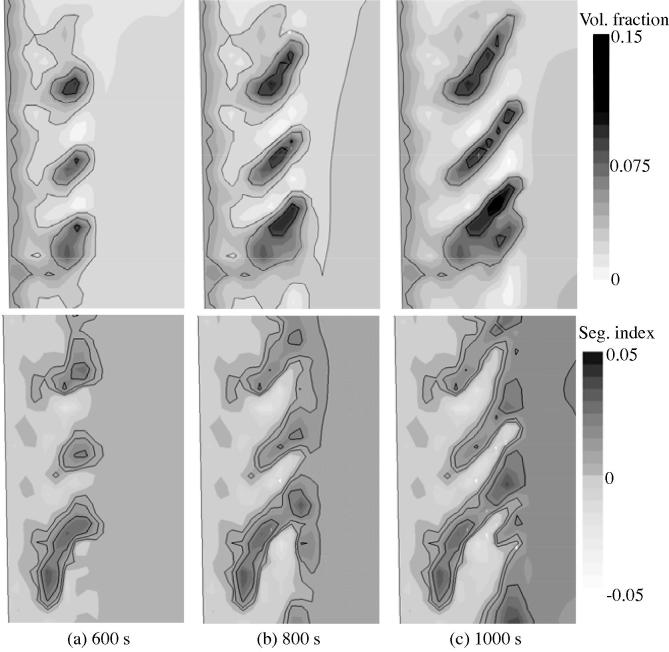
Zoomed-in results (Zone 2 is as marked in Fig. 7(d)) of the volume fraction of the equiaxed phase and the segregation index for the case of mixed columnar-equiaxed solidification. The evolution sequences are shown at 600 s, 800 s and 1000 s. The upper row shows the equiaxed volume fraction in greyscale overlapped with isolines at 0.03, 0.05 and 0.08. The bottom row shows the segregation index in greyscale overlapped with isolines at 0.005, 0.01 and 0.02.

Secondly, a streak-like distribution between columnar and equiaxed phases is caused by the initial unstable flow in the vicinity of columnar tip front by the interaction between the sinking equiaxed crystals and the growing columnar tip front and by the entrapment of the equiaxed crystals in the columnar trunks. This streak-like phase distribution coincides with the pattern of quasi A-segregates, as shown in Fig. 14. The accumulation of equiaxed phase results in negative segregation. The equiaxed-accumulated region approximately coincides with the region of negative segregation, and the equiaxed-lean (more columnar phase) region has a positive segregation. This coincidence further confirms that the periodical accumulation of equiaxed phase along the columnar tip front strengthens the quasi A-segregates.

### 6.3 Prediction of CET

Correct description of columnar-to-equiaxed transition (CET) belongs to an important part of calculation of macrosegregation. The features of the current model to track the columnar primary tip front and to predict CET considering both soft blocking and hard blocking mechanisms were evaluated previously by the current authors [19,25,34]. Although we are not able to quantitatively evaluate the CET based on the current ingot due to lack of sufficient experimental data, the comparison of the predicted CET and global macrosegregation pattern with the sulphur print and measured segregation distribution (Fig. 3) gives additional confidence in the predictive capability of the model with respect to the CET.

**Fig. 15:**
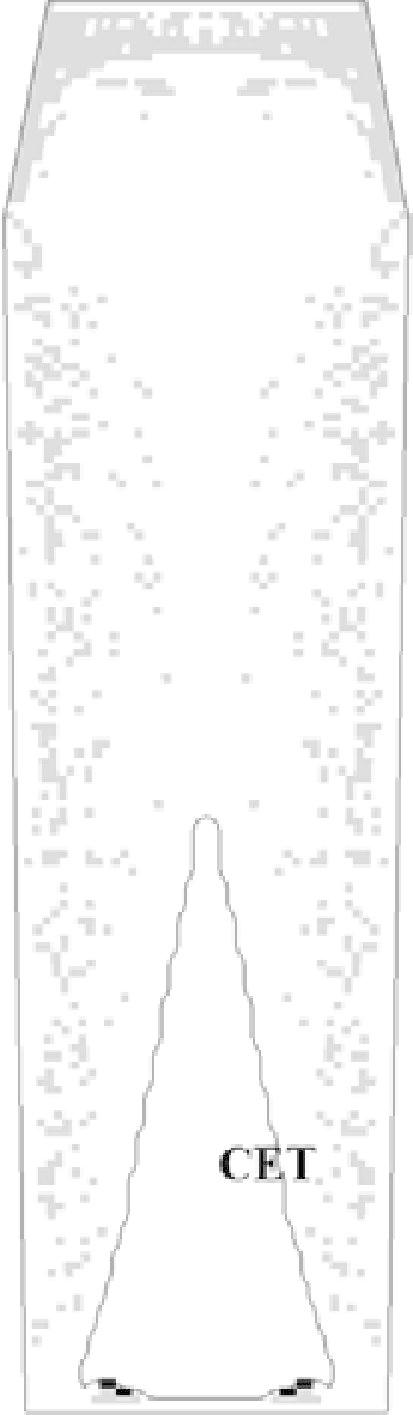
Analysis of the columnar tip blocking mechanism. The marked grey points are positions where the growth velocity of the columnar tip front is temporarily stopped (reduced to smaller than 10^−5^ m/s) by the mechanism of soft blocking. The black points are positions where the CET seems to be finally ‘frozen’ by the mechanism of soft blocking.

Fig. 15 records the positions where the mechanism of soft blocking operates in the ingot. It is interesting to find that the final CET line is mostly caused by the mechanism of hard blocking, except for a few elements in the bottom corners of the conic equiaxed zone where columnar tips seem to be finally ‘frozen’ by the mechanism of soft blocking. In the most cases, the growth of the columnar tips is only stopped temporarily by the mechanism of soft blocking (disappearance of constitutional undercooling). At the right moment the volume fraction of equiaxed phase in the front of columnar tips is smaller than 0.49 (hard blocking criterion). At the next moment if the constitutional undercooling develops again, the columnar tips can continue to grow. This mechanism of temporal soft blocking operates very frequently, in the middle and upper parts of the ingot. An additional point should be kept in mind is that the growth of equiaxed grains will stop as well, when the growth of columnar tips is soft-blocked. In other words, if one wants to ‘frozen’ the soft-blocking position of the columnar tips permanently, the local volume fraction of equiaxed phase should be allowed to increase to over 0.49, and this increase can only happen through transport of equiaxed phase from neighbourhood to the columnar tip front. This soft-blocking induced CET is only observed in the bottom corners of the conic equiaxed zone. To conclude the above discussion, both soft blocking and hard blocking are important, although the final CET line is mainly caused by the mechanism of hard blocking.

### 6.4 Uncertainties of the results

One novel feature of the chosen model is to incorporate three phases (liquid, equiaxed and columnar), but one drawback is that the calculation becomes exhausting. In order to consider the applicability of the model for large steel ingot, a compromise must be made between the model completeness and the computational cost. Concerns about uncertainties of the modelling results due to model simplifications and some recommendations for further model development are addressed as follows.

As we stated that the ignorance of dendritic morphology of equiaxed crystals leads to an overestimation of the intensity of bottom negative segregation. In practise, it was observed that grain morphology in fairy large steel ingots experiences a transition from globular (mostly in the bottom region) to dendritic (in the upper part of ingot) structure [14,35,36]. A study by Zaloznik and Combeau has shown that in the lower bottom globular region, the sedimentation is dominant for the negative segregation. In the quite dendritic equiaxed zone, the dendrites can pack very early, and the contribution of the settling effect on the macrosegregation can be ignorable and the main mechanism for the formation of the macrosegregation is the thermosolutal convection in the interdendritic region. Therefore, a further step incorporating dendritic morphology is desired. The authors have recently developed a 5-phase model [25,34] considering full dendritic morphology in both equiaxed and columnar phases. Due to the extremely high calculation cost, the model is currently applicable to a size of laboratory ingot [37]. Therefore, an intermediate solution might be applied. For example, the growth of the equiaxed dendritic envelope is treated according to KGT model [38], while the phase change is based on a Stefan problem assuming a spherical geometry of the solid phase inside the grain envelope [14,36].

In Section 4.2 we pointed out that the position and intensity of the positive segregation in the hot top region is not correct due to the ignorance of the solidification shrinkage. Some other consequences caused by this ignorance should also be addressed. One is the sinking top surface might adapt the thermal and flow boundary conditions there. Another important effect is on the interdendritic flow. Due to the solidification shrinkage, feeding flow might influence the shape and intensity of the quasi-A-segregation pattern as well. A new modelling step incorporating 4 phases (air, liquid, equiaxed and columnar) is currently undertaken, but more efforts are demanded.

Current paper used the locally linearized Fe–C binary phase diagram, but technical alloys are mostly multicomponent. Influence of other alloy elements on the segregation of carbon should be considered in two aspects. One is that the included alloy elements may alter the solidification path through thermodynamics and solidification kinetics [39,40]. Another aspect is that they may alter the density of the melt and influence the flow pattern through hydrodynamics [16,41]. A model incorporating multicomponent thermodynamics, diffusion-governed solidification kinetics and flow is still under development [13,42].

Appropriate estimation of the diffusion length is also an important issue for determining the diffusion-governed solidification path. In the current model, simple crystal morphology is assumed: sphere for globular equiaxed grain, and cylinder for columnar (cellular) trunks. The diffusion length in liquid phase is estimated by solving a diffusion field around a growing crystal (spherical or cylindrical) analytically [43]. The crystal is assumed to grow in a confined volume with a radius of *R*_f,e_ (or *R*_f,c_), which is determined according to the equiaxed grain number density (or the columnar primary dendrite arm spacing), hence the impingement of the solute distribution field at the end of solidification is treated. As shown in Eqs. (14)–(16), the liquid diffusion lengths are estimated *R*_e_(1 − *R*_e_/*R*_f,e_) and *R*_c_ ln(*R*_f,c_/*R*_c_) for equiaxed and columnar crystals, respectively. For dendritic solidification alternative methods should be used to calculate the diffusion length [44–47]. Flow and melt convection would modify the diffusion length as well [48,49].

## 7 Conclusion

A three-phase mixed columnar-equiaxed solidification model was applied to study the formation of macrosegregation in a 2.45-ton industry steel ingot. The predicted macrostructure and macrosegregation results agree qualitatively with the experimental data.

− Equiaxed sedimentation is a very important mechanism responsible for the formation of global segregation in such ingots. The calculation for solidification of both columnar and equiaxed phases possesses a segregation (index) range between −0.3 and 0.22; whereas the calculation for pure columnar solidification possesses a segregation range between −0.1 to 0.14. For the case of mixed columnar-equiaxed solidification, varying the equiaxed nucleation parameters changes the area of the lower conic negative segregation zone correspondingly, but the lower and upper limits of the global segregation index are only slightly influenced.
− Quasi A-segregates in the middle radius region between the casting outer surface and the centreline were predicted. Flow instability causes the formation of quasi A-segregates, but both the appearance of equiaxed crystals and their interaction with the growing columnar dendrites strengthen the segregates significantly. The equiaxed phase is not a necessary condition for the formation of quasi A-segregates because similar segregation bands with less severity are also found during pure columnar solidification.
− The discontinuous negative-positive segregation pattern under the hot top was predicted. This type of segregation is caused by the formation of a mini-ingot and by the subsequent sedimentation of equiaxed crystals within the mini-ingot.
− The calculated quantitative result of segregation is sensitive to the numerical grid resolution. A very fine grid is required to predict the details of quasi A-segregates. A grid-independent result regarding the fine details of quasi A-segregates in such ingots is not attainable based on current calculations. However, the global segregation pattern, e.g., the positive segregation extreme, negative segregation extreme, and their locations, can be obtained using a relatively coarse grid (10 mm).

The quantitative discrepancy between the calculations and the experimental results is still significant. The main reasons for this discrepancy from a modelling point of view are as follows: (1) over-simplification of the dendritic morphology of the crystals; (2) ignoring the solidification shrinkage; (3) simplification of the phase diagram; etc. To improve the accuracy of the calculations, further improvements in these areas are needed, in addition to reliable process conditions and more accurate material properties.

## Figures and Tables

**Table 1:** Conservation equations, sources and exchange terms, and auxiliary equations.

**Table 2:** Thermodynamic and physical properties.

Property	Symbols	Units	Quantity	Reference
Melting point of pure iron	*T*_f_	K	1805.15	[14]
Liquidus slope	*m*	K (wt.%)^−1^	−80.45	[14]
Equilibrium partition coefficient	*k*	–	0.36	[16]
Reference density	$\rho_{l}$, *ρ*_e_, *ρ*_c_	kg m^3^	6990	[14]
Solid–liquid density difference	Δ*ρ*	kg m^3^	150	[28]
Specific heat	$c_{\text{p}}^{l}\text{,}c_{\text{p}}^{\text{c}}\text{,}c_{\text{p}}^{\text{e}}$	J kg^−1^ K^−1^	500	[14]
Thermal conductivity	$k_{l}$, *k*_e_, *k*_c_	W m^−1^ K^−1^	34.0	[14]
Latent heat	*L*	J kg^−1^	2.71 × 10^5^	[14]
Viscosity	*μ*	kg m^−1^ s^−1^	4.2 × 10^−3^	[14]
Thermal expansion coefficient	*β*_T_	K^−1^	1.07 × 10^−4^	[14]
Solutal expansion coefficient	*β*_c_	wt.%^−1^	1.4 × 10^−2^	[14]
Dendritic arm spacing	$\lambda_{1}$	m	5 × 10^−4^	[14]
Diffusion coefficient (liquid)	$D_{l}$	m^2^ s^−1^	2.0 × 10^−8^	[29]
Diffusion coefficient (solid)	*D*_e_, *D*_c_	m^2^ s^−1^	1.0 × 10^−9^	[29]
